# Stable Supercapacitors Based on Activated Carbon Prepared from Italian Orange Juice

**DOI:** 10.3390/nano14010071

**Published:** 2023-12-26

**Authors:** Andrea Scarcello, Francesca Alessandro, Yolenny Cruz Salazar, Melvin Arias Polanco, Cristian Vacacela Gomez, Talia Tene, Marco Guevara, Stefano Bellucci, Salvatore Straface, Lorenzo S. Caputi

**Affiliations:** 1 Surface Nanoscience Group, Department of Physics, University of Calabria, 87036 Rende, Italy; 2 UNICARIBE Research Center, University of Calabria, 87036 Rende, Italy; 3 Institute on Membrane Technology, National Research Council of Italy (CNR-ITM), Via P. Bucci 17/C, 87036 Rende, Italy; 4 Laboratorio de Nanotecnología, Area de Ciencias Básicas y Ambientales, Instituto Tecnológico de Santo Domingo, Av. Los Próceres, Santo Domingo 10602, Dominican Republic; 5 INFN-Laboratori Nazionali di Frascati, 00044 Frascati, Italy; stefano.bellucci@lnf.infn.it; 6 Department of Chemistry, Universidad Tecnica Particular de Loja, Loja 110160, Ecuador; 7 Faculty of Mechanical Engineering, Escuela Superior Politécnica de Chimborazo (ESPOCH), Riobamba 060155, Ecuador; 8 Department of Environmental Engineering (DIAm), University of Calabria, Via P. Bucci, Cubo 42B, 87036 Rende, Italy

**Keywords:** orange juice, activated carbon, supercapacitor, electrodes, renewable materials

## Abstract

The development of efficient energy storage systems is critical in the transition towards sustainable energy solutions. In this context, the present work investigates the viability of using orange juice, as a promising and sustainable precursor, for the synthesis of activated carbon electrodes for supercapacitor technologies. Through the carbonization-activation process and controlling the preparation parameters (KOH ratio and activation time), we have tailored the specific surface area (SSA) and pore size distribution (PSD) of the resulting carbon materials—crucial parameters that support supercapacitive performance. Several spectroscopic, morphological, and electrochemical techniques are used to characterize the obtained carbon materials. In particular, our optimization efforts revealed that a 5:1 KOH ratio with an activation time up to 120 min produced the highest SSA of about 2203 m^2^/g. Employing these optimal conditions, we fabricated symmetric coin cell supercapacitors using Na_2_SO_4_ as the electrolyte, which exhibited interesting specific capacitance (~56 F/g). Durability testing over 5000 cycles sustained the durability of the as-made activated carbon electrodes, suggesting an excellent retention of specific capacitance. This study not only advances the field of energy storage by introducing a renewable material for electrode fabrication but also contributes to the broader goal of waste reduction through the repurposing of food byproducts.

## 1. Introduction

In the area of energy storage, supercapacitors have emerged as formidable candidates, supported by their distinct power density, rapid charge-discharge profiles, and sustained cycle durability [1,2,3]. These electrochemical storage systems predominantly segregate into two categories: electric double-layer capacitors (EDLCs) and pseudocapacitors [4,5]. The operational mechanism of EDLCs is anchored in the reversible adsorption of ions at the electrode-electrolyte interface, primarily facilitated by electrostatic forces, marking a non-faradaic process [6]. Conversely, pseudocapacitors rely on the expeditious and reversible redox transitions intrinsic to their faradaic energy storage mechanism [7]. These systems have been integrated across a myriad of fields, from vehicular applications [8] and next-generation electronic devices [9] to sustainable energy platforms [10], energy transducers [11], aerospace technologies [12], and nuanced electrochemical procedures [13].

In general, supercapacitors offer several advantages over traditional batteries [14,15], including remarkably rapid charge and discharge rates, exceptional cycle life with the capability to endure millions of cycles without significant degradation, higher power density for quick energy delivery, operational reliability across a broader range of temperatures, minimal maintenance requirements, and often, greater environmental safety due to the potential use of less harmful materials. These characteristics make supercapacitors ideal for applications that demand instant power bursts and high reliability, complementing the energy storage landscape alongside batteries.

Now, the primary advantage of EDLCs compared to pseudocapacitors is their exceptionally high power density and rapid charge/discharge capabilities, making them ideal for applications requiring quick energy bursts [16]. This advantage stems from their energy storage mechanism, which relies on electrostatic double-layer capacitance formed at the interface between the electrode material and the electrolyte, enabling rapid energy release within seconds [17]. In particular, improving the electrodes in EDLCs is essential to enhancing global performance. These improvements, centered on increasing electrode surface area, result in boosted capacitance and energy storage capacity. Moreover, optimized electrode materials lead to higher energy density, improved power density, extended cycle life, reduced internal resistance, enhanced environmental sustainability, and tailored performance for specific applications [18,19], rendering supercapacitors more versatile and efficient.

Electrodes in EDLCs are primarily composed of activated carbon (AC) because the porous structure of AC provides an extensive and accessible surface area, allowing ions in the electrolyte to be adsorbed in large quantities [20]. This results in higher capacitance and increased energy storage capacity. Furthermore, AC is chemically stable, ensuring long-term performance and reliability, while its low electrical resistance minimizes energy losses during charge and discharge cycles [21]. On the other hand, the tunability of AC properties, such as pore size and surface functionalization, allows manufacturers to customize electrodes for specific performance requirements.

AC can be obtained through the activation-carbonization process [22] using various carbon-rich sources, including agricultural waste and biomass [23,24,25,26]. An intriguing and underexplored avenue for producing AC is orange juice. However, the use of orange juice for this purpose has been limited, largely due to concerns about its role in the food supply. On the other hand, orange juice may seem like an inefficient carbon source, seemingly incapable of yielding significant quantities of AC. This idea could lead to the belief that large volumes of juice would be needed, potentially conflicting with the demand for this valuable beverage in the food industry. Nevertheless, a pressing issue exists, particularly in countries such as Italy [27], where tons of surplus oranges are wasted annually [28,29,30], posing significant environmental challenges in terms of disposal and management, which in turn demands innovative solutions. Hence, the conversion of surplus orange juice into AC presents a unique opportunity. By doing so, one can effectively address both the environmental concern of wasted fruit and the potential use of as-obtained AC for improved electrodes.

While there is extensive literature on the preparation of AC from orange peel [31], only a handful of studies have delved into harnessing carbon-based materials from orange juice for some applications. For instance, Angin et al. [32] explored the utilization of AC derived from fruit juice for Yellow 18 adsorption, while Sahu et al. [33] demonstrated the preparation of highly luminescent carbon dots from orange juice, proving their efficacy as exceptional bio-imaging agents. Recently, our group [26] successfully synthesized and characterized porous carbon materials from orange juice with a high specific surface and high density of mesopores through hydrothermal carbonization and potassium hydroxide (KOH) activation. We point out that these studies have not explored the production of AC and its potential application in supercapacitor technology. However, there is well-documented research on the use of hard carbons derived from banana stems and potato starch, which have been tested as supercapacitor electrodes and shown to exhibit high specific capacitance values [34].

In the context of orange juice, such missing investigation is presented here through comprehensive morphological, spectroscopic, and electrochemical characterization. Particularly, this study addresses, for the first time, the research gap on porous carbon samples obtained through variations in KOH ratio (3:1, 4:1, and 5:1) and activation residence time (30, 120, and 210 min), with similar specific surfaces but distinct pore size distributions (PSD). The obtained materials demonstrated exceptional stability over 5000 charge-discharge cycles and achieved a maximum specific capacitance of 56 F/g. Additionally, a higher specific surface area (SSA) of about 2203 m^2^/g was observed. Our findings highlight the potential of carbon materials synthesized from orange juice for supercapacitor technology, contributing to energy storage solutions.

## 2. Material and Methods

### 2.1. Materials

Oranges (*Citrus Sinensis*) were purchased from a local market in Rende, Italy. Chemicals, including potassium hydroxide (KOH, ACS reagent, ≥85%), hydrochloric acid (HCl, ACS reagent, 37%), sodium sulfate (Na_2_SO_4_, ACS reagent, ≥99.0%), carbon black (matrix Mesoporous Carbon, 45 μm), and 1-methyl-2-pyrrolidone (NMP, ACS reagent, ≥99.0%), were procured from Sigma Aldrich, St. Louis, MA, USA. Polyvinylidene fluoride (PVDF, 0.22 µm pore size, hydrophilic PVDF, 47 mm membrane) was acquired from Arkema, Colombes, France. All chemicals were used as received.

### 2.2. Preparation Process

The synthesis process is based on our previous work [34]. However, some obvious modifications were developed to improve the synthesis.

Freshly squeezed orange juice underwent filtration through a fine-mesh stainless sieve to extract the liquid from all solid residues. A total of 225 mL of filtered juice was introduced into a PTFE-lined stainless-steel autoclave with a 300 mL capacity, positioned within a lab stove, and subjected to heating at 180 °C for 6 h. Subsequently, the autoclave was gradually cooled to room temperature, and the resultant product was subsequently filtered and air-dried overnight at 80 °C, ultimately yielding 7.2 g of biochar. The obtained biochar was further subjected to treatment within a tubular furnace, where it was exposed to temperatures of 800 °C under an 800 mL/min nitrogen flux, yielding 3.6 g of pyrolyzed carbon. This pyrolyzed carbon was subsequently blended with KOH via an ultrapure water solution at varying KOH/carbon weight ratios. After water evaporation at 80 °C, the mixture was subjected to heating at 800 °C under an 800 mL/min nitrogen flux for varying residence times. The resultant product was then cooled to room temperature, subjected to washing with 1 M HCl to eliminate K-containing compounds, filtered using distilled water until a pH of 7.0 was attained, and finally dried overnight at 80 °C.

The AC samples were carefully prepared via mixing them with carbon black and polyvinylidene fluoride (PVDF) in a weight ratio of 85% AC, 10% carbon black, and 5% PVDF. This mixture was then stirred in N-methyl-2-pyrrolidone (NMP) solvent for 2 h at 60 °C to ensure thorough blending. To achieve a uniform consistency, the slurry was further homogenized using a mortar and pestle for 15 min. Subsequently, the homogenized slurry was applied to coat the current collectors of CR2032 coin cells, forming layers approximately 250 µm thick using a blade technique.

Following the coating process, the collectors were dried in a vacuum oven at 80 °C under a pressure of $2\times 10^{-5}$ mbar for 12 h to remove any residual solvent. In preparation for cell assembly, both collectors, along with a polyamide separator, were soaked in a 1 M Na_2_SO_4_ electrolyte solution. The mass loading on each electrode was meticulously controlled to be approximately 0.002 g, ensuring optimal performance due to the balanced composition that enhances both electrochemical performance and mechanical stability.

Let us stress again that this research employs Na_2_SO_4_ as an electrolyte to test activated carbon capacitance, prioritizing environmental sustainability and cost-effectiveness. The neutral pH of Na_2_SO_4_ reduces secondary reactions, ensuring accurate and reproducible results, while its lower ecological impact and cost align with the goals of green chemistry and scalable applications. This approach not only explores the electrochemical properties of activated carbon but also underscores a commitment to environmentally responsible research methodologies.

### 2.3. Characterization

The sample morphology was characterized using various techniques: transmission electron microscopy (TEM) was conducted on formvar-coated copper grids with an 80 kV acceleration voltage, employing a JEM 1400 Plus microscope (JEOL Ltd., Tokyo, Japan); scanning electron microscopy (SEM) was performed with a 5 kV acceleration voltage, utilizing an EVO 10 microscope (Carl Zeiss AG, Oberkochen, Germany); X-ray photoelectron spectroscopy (XPS) measurements were carried out in an ultra-high vacuum system equipped with a Phoibos 100 hemispherical analyzer (SPECS HSA 3000 plus), using non-monochromatized Mg-kα radiation; Raman spectra were recorded using an NRS-500 spectrometer (Jasco Corp., Tokyo, Japan) with a 532 nm laser wavelength (0.3 mW, 100× objective) on samples deposited onto a glass substrate; nitrogen adsorption/desorption isotherms were acquired using an ASAP2020 Plus system (Micromeritics Instrument Corp., Norcross, GA, USA) at liquid nitrogen temperature (−196 °C), and specific surface area (SSA) and pore size distribution (PSD) were determined through the Brunauer–Emmett–Teller (BET) and non-local density functional theory (NLDFT) methods, respectively; finally, coin-cell supercapacitors were tested using an Autolab PGSTAT302n potentiostat (Metrohm Autolab BV, Utrecht, The Netherlands).

## 3. Results and Discussions

### 3.1. Brunauer–Emmett–Teller (BET) Analysis

As previously stated, the performance of supercapacitors is intrinsically reliant upon the electrochemical characteristics of their electrodes, with specific emphasis placed on attributes encompassing SSA, pore size distribution, and electrical conductivity [35]. Specifically, electrodes characterized by large SSAs find their utility in various supercapacitor configurations.

In addition, it is imperative to underscore that the choice of electrolyte represents an essential factor in supercapacitor performance. For instance, sulfuric acid (H_2_SO_4_) is traditionally favored for its exceptional ionic conductivity and broad operational voltage range, contributing to superior power and energy densities. Nevertheless, sodium sulfate (Na_2_SO_4_) is emerging as a valuable alternative due to its notable safety advantages [36]. Being non-corrosive, Na_2_SO_4_ minimizes operational risks and concerns related to leakage, thereby promoting a safer working environment. Its adoption also leads to improved cycling stability, which extends the lifespan and ensures consistent supercapacitor performance [37]. Furthermore, its compatibility with supercapacitor materials reduces the rate of component degradation, further enhancing the longevity of the device. Although Na_2_SO_4_ may confer a higher energy density under certain conditions, it maintains a manageable operational temperature range, making it a practical choice for a broad spectrum of applications.

Considering these points, the present study opts for Na_2_SO_4_ as the electrolyte while also seeking an electrode material characterized by a substantial SSA. To achieve this goal, a series of experiments have been conducted (triplicate), exploring two important preparation parameters to strike the right balance between activation time (ranging from 30 min to 210 min) and the KOH ratio (ranging from 3:1 to 5:1). These systematic investigations are geared towards optimizing supercapacitor performance to its fullest potential. Additionally, we have explored KOH ratios up to 8:1 (results not shown here). Our data indicate a trend towards saturation beyond a 5:1 ratio. Therefore, the plateau in the SSA implies that higher KOH ratios may not be cost-effective or environmentally sensible, given that the benefits do not justify the increased costs and potential negative impacts.

Table 1 reveals that at a lower KOH ratio of 3:1, the SSA remains below approximately 1780 m^2^/g, even with extended activation times (up to 210 min). However, as the activation time increases, so does the SSA. At a KOH ratio of 4:1, the SSA increases up to 120 min of activation time, peaking at approximately 2196 m^2^/g. Interestingly, this value decreases to around 1574 m^2^/g at 210 min, likely due to over-activation. Similarly, with a KOH ratio of 5:1, the SSA increases with longer activation times, reaching a maximum of about 2203 m^2^/g at 120 min. Given these remarks, our subsequent analyses in this paper will primarily focus on samples activated at 4:1@120 (with an SSA of 2195.85 ± 7.66 m^2^/g), 5:1@120 (with an SSA of 2202.81 ± 7.43 m^2^/g), and 5:1@210 (with an SSA of 1926.69 ± 7.42 m^2^/g), as they exhibit the highest SSAs among the tested conditions.

**Figure 1:**
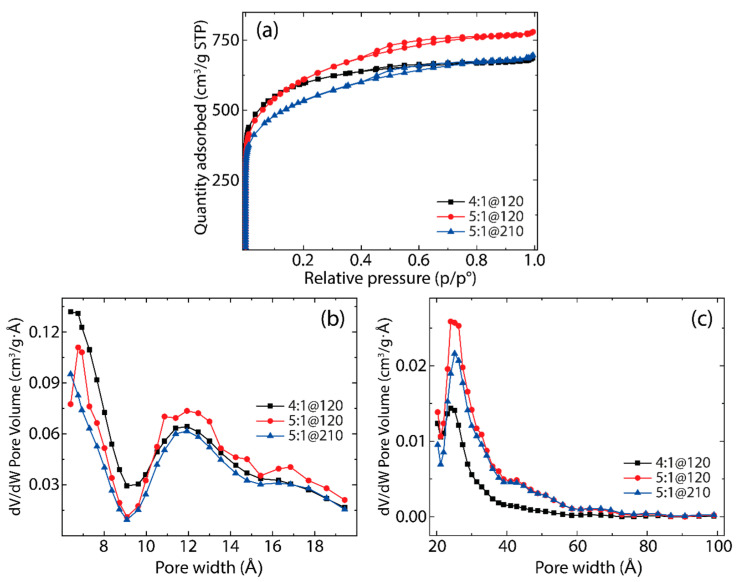
(**a**) Nitrogen sorption isotherms of the active carbons, showing some hysteresis. Pore size distributions were obtained using the NLDFT method from (**b**) 6 to 20 Å and from (**c**) 20 to 100 Å.

To further emphasize, Figure 1a presents the nitrogen (N_2_) adsorption-desorption isotherms for the three samples that exhibited the largest SSAs, as determined using the BET model (for all synthesized samples, refer to Figure S1a). These isotherms serve as a tool in discerning the effects of activation parameters on the pore structure and pore size distribution (PSD) of the carbon-based materials under investigation. The observed isotherms, which align closely with the characteristics of Type VI, display a pronounced uptick at relative pressures below 0.5, indicative of the prevalent microporosity across all samples. Moreover, the hysteresis loop discernible between P/P^0^ values of 0.5 and 0.8 reveals the existence of mesopores [38]. This mesoporosity is especially prominent in samples 5:1@120 and 5:1@210, while it appears to be subdued for the 4:1@120 sample. These observations align with the pore size distribution curves depicted in Figure 1b,c, calculated from Non-Local Density Functional Theory (NLDFT) analysis. Furthermore, the reliable presence of hysteresis across all samples indicates the composition of the electrode material, potentially featuring a combination of slit-like and cylindrical pores [39]. These insights underline the careful selection of these carbon materials as potential candidates for the fabrication of high-performance supercapacitor prototypes.

Figure 1 and Figure S1b highlight the significant impact that activation parameters induce on the PSD. For the sample with a KOH ratio of 4:1 and an activation time of 120 min (4:1@120, black curve), the primary constitution of the material leans towards nanopores, as shown in Figure 1b. However, this sample exhibits a subdued presence of micro- and mesopores, as depicted in Figure 1c. On adjusting the KOH ratio to 5:1 and maintaining an activation time of 120 min (5:1@120, red curve), the resultant material displays a well-distributed formation of nano-, micro-, and mesopores. The pore size variations become even more pronounced for the sample with a KOH ratio of 5:1 and an extended activation time of 210 min (5:1@210, blue curve). In this material, the intervening walls separating the pores undergo further degradation, confirming the decline in the SSA [40] (Table 1). Notably, the 5:1@210 sample exhibits a reduction in both nano- and mesopore quantities when contrasted with the 4:1@120 and 5:1@120 samples, respectively. Taking these outcomes into account, the most interesting balance between meso- and micropores is observed for the 5:1@120 sample. This balance infers an accessible surface area favorable for ions, facilitating the creation of a double layer and promoting electrolyte permeation within the electrode material. Such characteristics are essential in determining the global efficiency and performance of supercapacitors [41].

We point out that while SSA is an important parameter for supercapacitors, it is not the only determinant of capacitance. The selection of the top three samples for detailed analysis was based not solely on SSA, but also on their electrochemical performance, which includes factors such as pore structure and electrical conductivity. In addition, preliminary tests confirmed that these samples exhibited the most balanced properties for supercapacitor functionality, thus justifying their advancement to further stages of evaluation.

### 3.2. Morphological Analysis

The preparation of AC is a multi-faceted process that encompasses stages like hydrothermal treatment—a method that aids in the transformation of biomass precursors into carbon-rich structures. Alongside this, the hydrolysis of carbohydrates simplifies complex biological materials, setting the stage for subsequent carbonization and activation. Notably, these procedures can induce the creation of microspheres, a phenomenon driven by nucleation and subsequent growth mechanisms [42]. Such details in preparation ensure that the AC achieves a tailored structure and porosity, optimizing it for various applications.

Taking this into account, Figure S2 provides SEM images of a pyrolyzed sample, highlighting the microstructure morphology. In Figure S2a, we observe a dense agglomeration of near-microspherical particles, uniformly distributed and closely packed together. The overall surface appears to be fairly homogenous, with particles demonstrating relatively consistent sizes. In Figure S2b, the SEM image offers a close-up view of an individual sphere, capturing its smooth, almost featureless surface. The detailing makes it evident that the sphere has a diameter of approximately 5 μm. This higher-resolution image helps in appreciating the integrity and uniformity of each microsphere, reinforcing their consistency in size and shape. Following the pyrolysis process and before activation, the microspheres displayed a size distribution with a mean diameter of 6.6 μm and a standard deviation of 1.5 μm (Figure S3).

**Figure 2:**
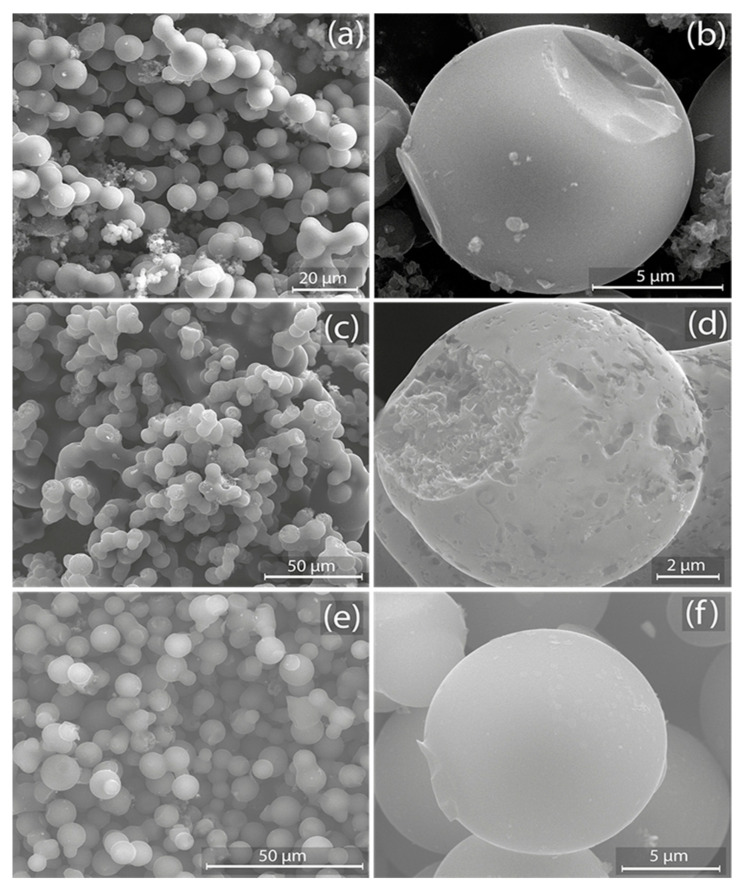
SEM images of activated carbon samples: (**a**,**b**) 4:1@120, (**c**,**d**) 5:1@120, and (**e**,**f**) 5:1@210.

Figure 2 presents SEM images of AC samples. Specifically, Figure 2a (4:1@120) depicts a collection of tightly packed microspheres with evident clustering. In a magnified view, Figure 2b reveals a large microsphere characterized by diminutive protrusions and adhered particles, boasting a diameter close to 5 μm. Figure 2c (5:1@120) illustrates a morphology that is more clustered and heterogeneous, with microspheres of varied sizes and irregular shapes. Figure 2d offers an up-close perspective of a microsphere, highlighting its rough and textured surface adorned with pits, crevices, and potential pore openings, confirming an increased surface area (see Table 1). Figure 2e (5:1@210) exhibits microspheres that seem more uniformly sized and evenly distributed than previous samples. Lastly, Figure 2f uncovers a microsphere with a notably smoother and more refined surface, a feature indicative of a reduced SSA (see Table 1). These results emphasize the significant impact of preparation conditions on the morphology of the as-made AC, underlining the relationship between preparation conditions and the resulting material.

**Figure 3:**
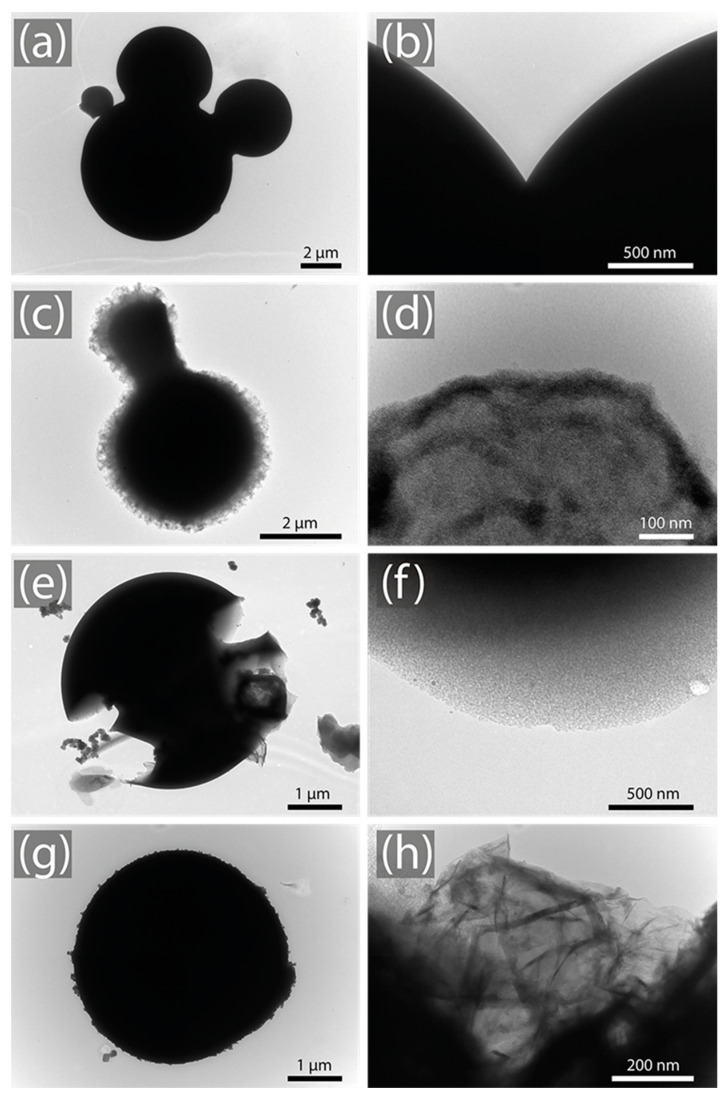
TEM images of (**a**,**b**) pyrolyzed sample, (**c**,**d**) 4:1@120, (**e**,**f**) 5:1@120, and (**g**,**h**) 5:1@210.

Figure 3 presents a series of TEM images elucidating the structural transitions across various samples. In Figure 3a,b, corresponding to the pyrolyzed sample, the pronounced high-contrast boundaries, especially in Figure 3a, highlight a notably dense periphery. This increased density is a consequence of the pyrolysis process, making it challenging for the electron beam of the microscope to penetrate, thus rendering the striking contrast. Figure 3c,d represent the 4:1@120 sample. Specifically, Figure 3c portrays the somewhat jagged and irregular contours of the sample. Figure 3d, taken at a greater magnification, accentuates the diminished density of the sample, revealing a distinctly porous morphology. The reduced contrast hints at this lower density, which results from the activation process.

Figure 3e,f show the analysis of the 5:1@120 sample, which manifest even more pronounced alterations. Figure 3e delivers a broader perspective of the spherical structure and underscores considerable signs of wear and tear, almost akin to erosion. Meanwhile, Figure 3f offers a closer view of the sample surface, and the diminished image contrast signifies a more porous and less dense structure. The pronounced jaggedness and irregularities, evident at this magnification, suggest significant changes in the structural composition of the sample, likely due to the longer activation time.

Figure 3e,f delve into the intricacies of the 5:1@120 sample, revealing more striking modifications. Figure 3e provides an expansive view of the spherical form, highlighting evident signs of degradation reminiscent of erosion. In contrast, Figure 3f zooms in on the sample surface. The notable reduction in image contrast indicates a structure that is both more porous and less dense. The manifest jaggedness and irregularities at this magnification allude to considerable shifts in the sample surface morphology, potentially attributable to extended activation times. Figure 3g,h analyze the 5:1@210 sample, particularly, Figure 3g portrays a relatively smooth and rounded structure, while Figure 3h, taken at a finer scale, unveils a complex, folded, and layered structure, reminiscent of a graphitized material.

### 3.3. Spectroscopic Analysis

**Figure 4:**
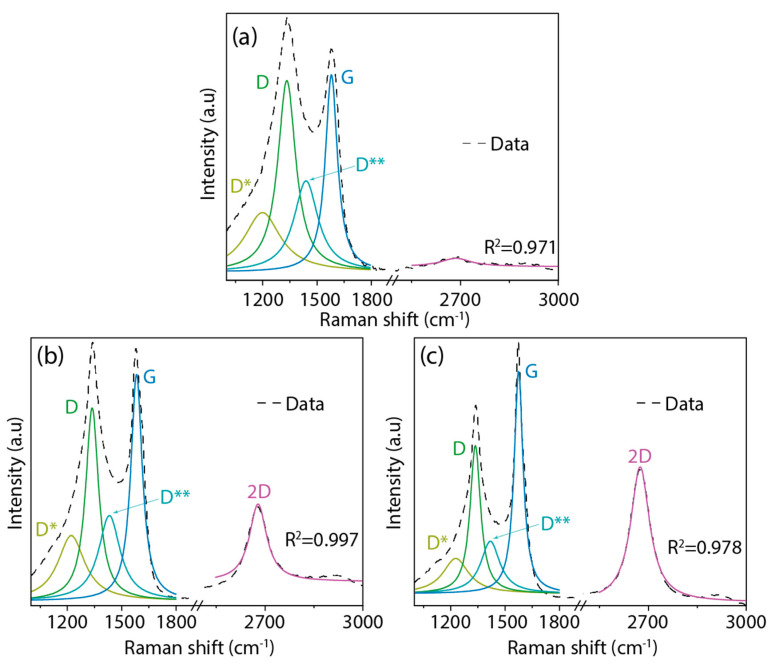
Raman spectra of (**a**) 4:1@120, (**b**) 5:1@120, and (**c**) 5:1@210. The intensity was normalized using the most intense peak and the data were fitted using Lorentzian functions.

We now focus on the Raman spectroscopy analysis of the AC samples (Figure 4). The specific peak positions have been detailed in Table S1. Each sample displays four distinct bands within the 1000 to 2000 cm^−1^ range. Predominantly, the D band is centered around 1334 cm^−1^ and the G band is close to 1578 cm^−1^. We also notice less intense bands: the D* band at approximately 1220 cm^−1^ and the D** band at around 1430 cm^−1^. Moving to the 2000 to 3000 cm^−1^ region, the 2D band emerges, a characteristic feature of graphitic-like materials.

Based on our prior work [43], the D band appears due to the presence of disordered structures within the carbon material and any defects in its graphitic-like structure. This band primarily indicates sp^3^ hybridized carbon atoms and irregularities within the carbon lattice structure. The G band, on the other hand, suggests graphitic-like configurations. This G band arises due to the E_2g_ phonon of sp^2^ hybridized carbon atoms in a two-dimensional hexagonal lattice, underscoring the graphitized nature of the obtained material. The D** band owes its presence to various factors, including the phonon density in finite-sized graphitic crystals, C–H vibrations in hydrogenated carbon, and hopping-like defects [44]. While the D* band is indicative of the sp^3^ diamond-like configuration on disordered amorphous carbons, we point out that the expansive region between approximately 1400 cm^−1^ and 1650 cm^−1^ does not correspond to diamond carbon phases [45]. Lastly, the 2D band, being the second harmonic of the D peak, is typically representative of few-layer graphene-like structures (Figure 4c), a fact further substantiated via our TEM findings (see Figure 3h).

To gain insight into the defect structures of the AC samples, we utilized the intensity ratios of the D and G bands (Table 2). This ratio serves as a reliable indicator of the degree of disorder or defects within the carbon structure. Moreover, we leveraged the intensity ratios of the 2D and G bands to provide an understanding of the layered structures within the samples (Table 2). A closer look at the data reveals that as the KOH ratio increases, the intensity ratio (I_D_/I_G_) diminishes, transitioning from 0.97 for the 4:1@120 sample to 0.68 for the 5:1@210 sample. This downward change implies a decrease in defects or an enhancement in the graphitization of the sample. Concurrently, the 4:1@120 sample lacks a discernible 2D band, leading to an intensity ratio (I_2D_/I_G_) of zero. In contrast, the subsequent samples, as they are subjected to increased KOH concentrations, exhibit a rise in the I_2D_/I_G_ ratio. This uptick corroborates the emergence of a layered carbon structure, further proven via the observations in Figure 3h. In essence, the increasing KOH ratio not only aids in diminishing defects but also promotes the formation of layered carbon structures, making the samples progressively more graphitic.

**Figure 5:**
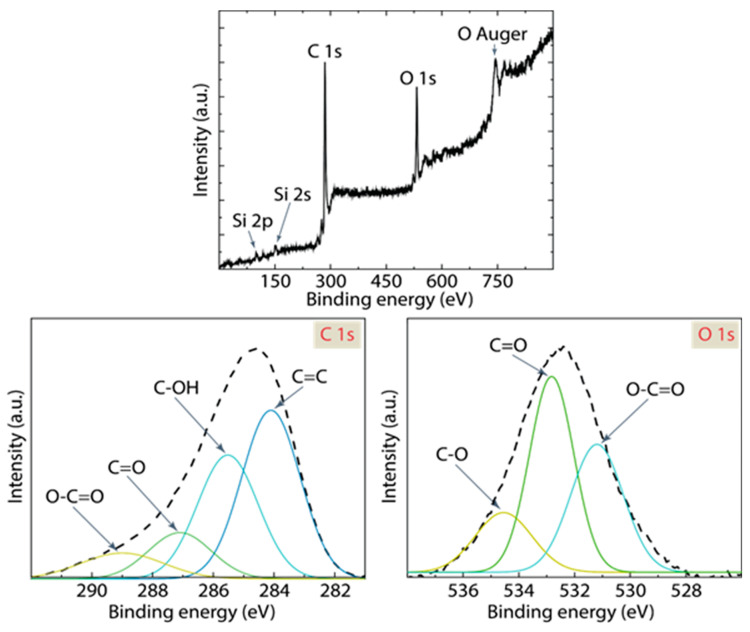
XPS spectrum of 5:1@120. Data was fitted using Gaussian functions.

X-ray photoelectron spectroscopy (XPS) provides in-depth insight into the elemental composition, chemical state, and electronic state of the elements present in a material [46]. From Figure 5 and Table 3, a detailed understanding of the surface chemistry of the 5:1@120 AC sample can be extrapolated. The other two samples (i.e., 4:1@120 and 5:1@210) show similar trends, not shown here. In Figure 5a, the broad XPS spectrum displays prominent peaks attributed to C 1s, O 1s, Si 2s, and Si 2p. The clear presence of these peaks confirms the existence of carbon, oxygen, and silicon (from the substrate) on the AC material. Notably, the C 1s and O 1s peaks are of significant intensity, emphasizing their predominant role in the sample chemistry. An additional feature worth mentioning is the “O Auger” peak, representing an Auger electron transition specific to oxygen, reinforcing the presence of oxygen-containing functional groups.

A close view into the C 1s region (Figure 5b), multiple peaks are resolved, representing different carbon functional groups: The peak at ~284.10 eV, attributed to C=C, indicates the presence of graphitic or sp^2^ hybridized carbon. This component is the most dominant, comprising 46% of the C 1s peak (Table 3). The C-OH peak at ~285.52 eV signifies hydroxyl functionalities, contributing to 34% of the C 1s spectrum. The C=O peak at ~287.10 eV, accounting for 13%, indicates carbonyl or quinonic functionalities. The O-C=O peak at ~289.00 eV, which is the least intense among the four, suggests carboxylic acid groups, contributing to 7% of the C 1s spectrum.

In the O 1s region (Figure 5c), the C-O peak at ~534.55 eV is the most dominant, accounting for 55% of the O 1s spectrum, highlighting that the material is rich in oxygen functional groups. The C=O peak at ~532.82 eV corresponds to 30%, further indicating the presence of carbonyl functionalities. The O-C=O peak at ~531.19 eV accounts for 15%, underscoring the carboxyl content in the sample.

In general, the XPS data suggests a rich presence of oxygen functional groups in the AC sample. The dominant C=C peak in the C 1s spectrum aligns with the inherent graphitic nature of AC as observed in Figure 4b. Concurrently, the significant contributions from C-OH, C=O, and O-C=O peaks highlight the chemically functionalized surface of the resulting material, which can be attributed to the activation process. The high R^2^ values of 0.999 for both C 1s and O 1s peaks emphasize the accuracy of the Gaussian fit, reinforcing the reliability of the functional group quantification provided in Table 3.

The crystalline structure of the biochar sample, both pre- and post-carbonization, was analyzed through X-ray diffraction (XRD) patterns. Before carbonization, the XRD pattern revealed predominantly noise, lacking any distinct peaks, indicative of an amorphous carbon structure. Post-carbonization, as depicted in Figure S4, a pronounced peak at approximately 22 degrees is noticeable. This peak corresponds to the (002) lattice plane, characteristic of graphite structures, suggesting the formation of graphite-like crystallinity in the carbonized biochar. This observation aligns with the increased carbon content and potential enhanced thermal stability of the sample, which is consistent with earlier characterization techniques reported in our study.

Additionally, since we do not have access to this type of study, we would like to suggest incorporating Thermogravimetric Analysis (TGA) in future studies to further complement the understanding of biochar as an electrode material for supercapacitors. TGA offers invaluable insights into the thermal stability, pore structure, and surface characteristics of biochar, all critical elements influencing electrode performance. This analytical technique is useful to evaluate the biochar performance across various temperature ranges, assessing its impact on capacitance, cycle stability, and global safety. Moreover, TGA can assist in detecting any impurities or additives present, which might influence supercapacitor functionality. Such comprehensive analysis is fundamental for refining the design and operational parameters of supercapacitors, ensuring their optimal efficiency and long-term durability.

We point out that the incorporation of oxygen functional groups into AC electrodes significantly influences their electrochemical behavior, particularly in the context of EDLCs. These oxygen groups enhance the hydrophilicity and wettability of the electrodes, a feature that is crucial for efficient electrolyte penetration into the porous structure of AC. Improved wettability ensures more effective ion transport, a key factor in optimizing non-Faradaic processes where swift charge/discharge kinetics are highly desirable. Furthermore, the presence of oxygen functional groups imparts additional surface charges to the electrodes. These charges facilitate electrostatic interactions with the ions present in the electrolyte at the electrode-electrolyte interface, which is an important aspect of the operational efficiency of EDLCs. Such interactions boost the storage capacity and the global energy efficiency of the capacitors.

However, the interaction dynamics between these functional groups and the electrolyte ions, particularly considering the size of the ions and their accessibility to the electrode pores, are complex. While it is challenging to precisely quantify their contribution to specific capacitance, it is evident from both our study and existing literature that the efficacy of these oxygen groups is closely tied to the pH of the electrolyte. In acidic or alkaline environments, the reactions of these functional groups with the electrolytes are more pronounced, thereby enhancing the capacitor performance. On the contrary, in neutral electrolytes like Na_2_SO_4_, the impact of these functional groups is more restrained. This variance underscores the need for a strategic selection of electrolytes in supercapacitor design, ensuring that the interaction between the electrolyte and oxygen-doped AC electrodes is optimized for maximum efficiency and performance.

### 3.4. Electrochemical Analysis

Three distinct AC materials were utilized to construct electrodes for CR2032 coin cell supercapacitors, exploiting the unique properties of each carbon type. The electrochemical characterization of these supercapacitors was carried out through cyclic voltammetry (CV) and constant current galvanostatic charge/discharge (CCGCD) techniques.

For the CV analysis, a potential range was selected from 0 to 1.4 V to thoroughly probe the electrochemical windows of the materials, with scan rates varying from a slow 0.001 V/s to a faster 0.1 V/s. This range of scan rates was chosen to explore the kinetics of ion intercalation and surface redox reactions within the electrodes.

The total capacitance ($C_{T}$), determined through the analysis of the voltammogram, is estimated as follows:

(1) $$C_{T}=\frac{\int_{0}^{2V_{0}}i\left(V\right)dV}{2\;r\;V_{0}}$$

Here, the integral bounds from 0 to $2V_{0}$ encompasses the entire area under the CV curve for both anodic (charging) and cathodic (discharging), $i\left(V\right)$ presents the current response at a given voltage V, r represents the scan rate (V/s), and $V_{0}$ is the potential range over which the CV is performed. The factor of $1/2\;r\;V_{0}$ normalizes the integral to account for the scan rate and the potential window, ensuring that the calculated capacitance is independent of test parameters. The factor of 2 is particularly important as it accounts for the double-layer charging and discharging process, effectively considering both the anodic and cathodic scans as one capacitive contribution rather than two separate events.

The specific capacitance ($C_{s}$) in farads per gram (F/g) is estimated using the following expression:

(2) $$C_{s}=\frac{C_{T}}{2m}$$

where m is the mass of the active electrode material. Since the device is symmetrical and equipped with two electrodes, each with mass m, the total active mass is 2m. This factor ensures that the specific capacitance is normalized to the mass of both electrodes combined, which is important for comparing the performance of different supercapacitor materials.

**Figure 6:**
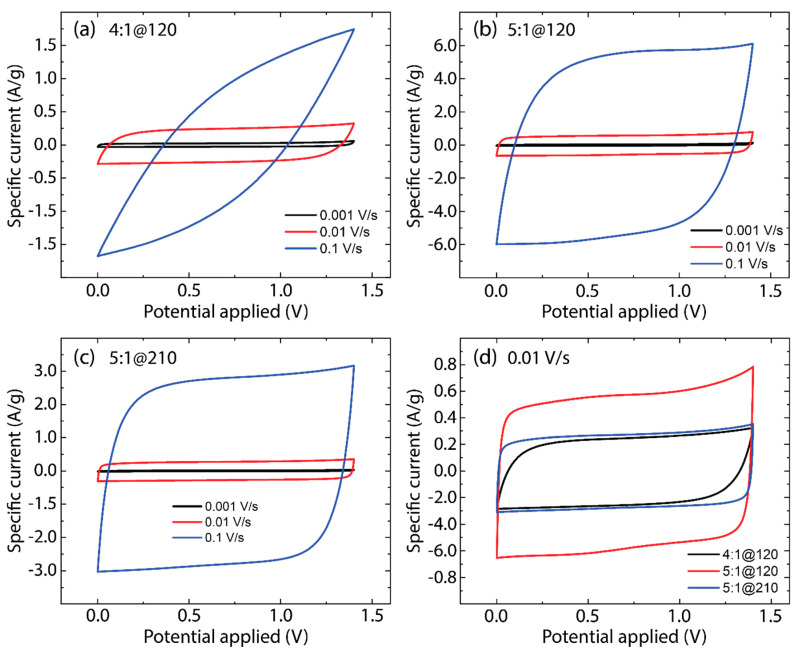
Cyclic voltammetry tests of (**a**) 4:1@120, (**b**) 5:1@120, and (**c**) 5:1@210. (**d**) Comparison of cyclic voltammetry diagram at 0.01 V/s.

Figure 6 displays the CV test at different scan rates (0.001 V/s, 0.01 V/s, and 0.1 V/s) for the as-made AC samples: 4:1@120 (Figure 6a), 5:1@120 (Figure 6b), and 5:1@210 (Figure 6c). The CV curves of supercapacitors using 5:1@120 and 5:1@210 electrodes show a nearly rectangular shape at lower scan rates (0.001 V/s and 0.01 V/s), suggesting ideal capacitive behavior with low equivalent series resistance (ESR) [34]. This indicates efficient ion transport and charge storage mechanisms within the electrode porous structure. Instead, the supercapacitor with 4:1@120 electrodes shows a departure from the rectangular shape at a lower scan rate (0.005 V/s), indicating a higher ESR, which could be due to less optimal material properties, such as lower graphitization levels, as observed in Raman results (see Figure 4a).

It is noteworthy that the more rectangular the CV curve, the closer the behavior is to an ideal supercapacitor, which is characterized by rapid charge-discharge processes and minimal resistive losses. Deviations from the rectangular shape at higher scan rates are normal due to the ESR of the supercapacitor, which includes resistance from the electrodes, separator, current collectors, and the electrolyte. The influence of ESR is particularly critical in determining the supercapacitor power density, as it dictates the rapidity with which energy can be transported and received by the device.

In Figure 6d, CV curves at a scan rate of 0.01 V/s are compared for all AC samples, revealing distinct capacitance values: 22.26 F/g for 4:1@120, 56.05 F/g for 5:1@120, and 26.95 F/g for 5:1@210. The comparatively lower capacitance observed for the 4:1@120 and 5:1@210 samples could be due to factors like less effective pore structure for ion intercalation, lower electrical conductivity, or poorer contact between the active material and the current collector. In contrast, the 5:1@120 sample demonstrates superior electrochemical performance with a specific capacitance of 56.05 F/g, emphasizing its efficiency and superior charge storage capacity. Such excellent performance is likely due to balanced synthesis parameters, including KOH ratios and activation time, which result in a microstructure optimized for electrochemical reactions. These findings stress the critical impact of the synthesis process on the properties of AC electrodes, affirming that the material preparation is crucial to advancing supercapacitor technology.

**Figure 7:**
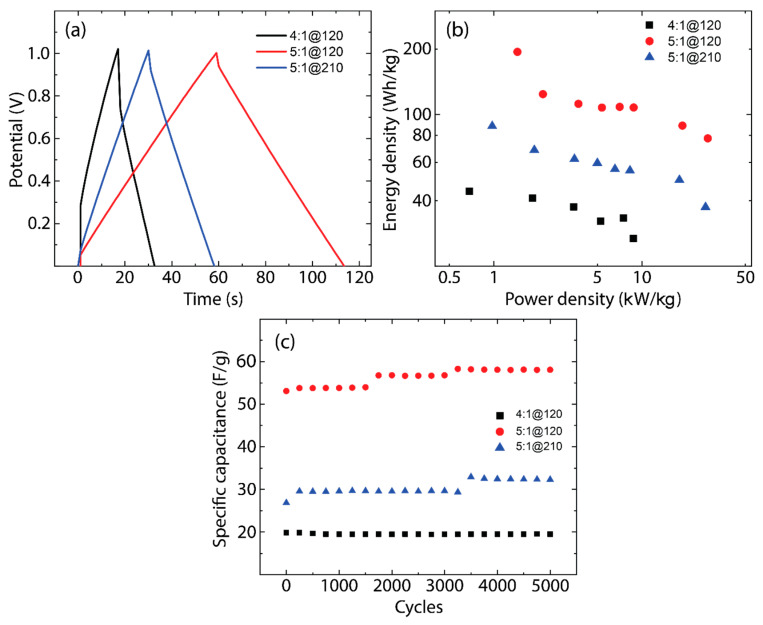
(**a**) Galvanostatic cycles, (**b**) Ragone plots, and (**c**) durability measurements for the three different samples: 4:1@120 (black curve), 5:1@120 (blue curve), and 5:1@210 (red curve). The durability test is carried out at a specific current of 1 A/g.

CCGCD cycles were performed over a range of specific currents from 0.10 A/g to 5.00 A/g, with the maximum voltage set at 1 V. The charge/discharge profile at 1.00 A/g, depicted in Figure 7a, exhibits an almost perfectly linear relationship between voltage and time, which is characteristic of ideal capacitive behavior. To quantify the device capacitance under these conditions, the following expression is used:

(3) $$C_{s}=\frac{i\;\Delta t}{\Delta V}$$

where, $C_{s}$ is the specific capacitance, i is the applied specific current, and Δt is the elapsed time for a given potential change, ΔV. It is critical to note that the capacitance values derived from these galvanostatic tests align with those obtained from cyclic voltammetry, providing a cohesive and reliable picture of the supercapacitor performance. This consistency across different testing methods reinforces the validity of the experimental results and the supercapacitor potential for practical application, given its predictable and stable behavior under varying electrical loads.

The array of specific current cycles ranging from 0.10 A/g to 5.00 A/g (i.e., 8 points: 0.10, 0.25, 0.50, 0.75, 1.00, 1.25, 3.00, and 5.00 A/g) served for calculating both the energy and power densities of the supercapacitors, which were important for plotting Ragone diagrams. These diagrams are depicted in Figure 7b, which provides a visual representation of the energy and power relationship of the devices. In particular, the data from the 4:1@120 supercapacitor exhibited notable voltage drops at the higher specific currents of 3 A/g and 5 A/g.

Based on CCGCD diagrams, the energy density and power density can be expressed using the following equations:

(4) $$Energy\;density=\int_{0}^{Q}V_{0}dq=\frac{1}{2}V_{0}Q=\frac{1}{2}V_{0}\;i{\;t}_{c}$$

and

(5) $$Power\;density=\frac{Energy\;density}{t_{d}}$$

where $V_{0}$ is the maximum voltage to charge the supercapacitor, Q is the net charge accumulated by the device, i is the constant current applied during the charge/discharge process, and ${\;t}_{c}$ is the charging period. Equation (4) is normalized with an appropriate parameter and a factor of 3600 yields, resulting in watt-hour units. In Equation (5), $t_{d}$ is the discharge time.

As noted, in CV tests, a 0–1.4 V range is selected to comprehensively assess the electrochemical behavior of the electrodes and to detect any faradaic reactions, such as redox activities or electrolyte breakdown, that might arise beyond the normal operating scope. This extended range also serves to establish the upper potential threshold, ensuring that subsequent tests operate within a regime that prevents material damage and safety hazards. Conversely, CCGCD tests utilize a more conservative voltage span of 0–1 V, chosen to guarantee stable and secure supercapacitor operation through steering clear of risks unearthed in CV analysis. This approach adheres to realistic operational conditions, crucial for safeguarding supercapacitor integrity and enhancing cycle life, particularly for rigorous durability assessments.

With this in mind, to assess the long-term stability and endurance of the supercapacitor prototypes, durability tests were conducted using galvanostatic charge/discharge cycling. Each prototype underwent a regimen of 5000 cycles at a specific current of 1 A/g within a potential range of 0 to 1 V. To monitor the performance degradation over time, the gravimetric-specific capacitance was evaluated every 250 cycles. The outcomes of these evaluations, which are critical indicators of the supercapacitor longevity and practical viability, are systematically illustrated in Figure 7c which provides insight into the capacitance retention of the supercapacitors. Specifically, the 4:1@120 sample (black markers) exhibits a marked decline in specific capacitance over 5000 cycles, indicating rapid degradation and suggesting potential issues with electrode material stability or electrolyte compatibility. In contrast, the 5:1@120 sample (red markers) maintains its specific capacitance more effectively, implying superior durability and suggesting more robust electrode integrity under repeated cycling. Meanwhile, the 5:1@210 sample (blue markers) starts with a higher specific capacitance, possibly due to a more favorable pore structure or greater active surface area, but it also shows a decline, hinting at stability trade-offs despite an initially higher charge storage capacitance.

All these trends are critical for assessing supercapacitor performance, as they indicate the ability of the materials to withstand long-term cyclic stress, a key factor for their application in energy storage solutions. In this context, we have conducted extra SEM and TEM analyses of the 5:1@120 samples after the maximum long-term cycling tested here (5000 cycles). Figure S5 shows an unchanged morphology of the AC sample after 5000 cycles, which signifies excellent structural and electrochemical stability, suggesting that the AC electrodes can endure extensive cycling without physical degradation.

### 3.5. Electrochemical Impedance Spectroscopy (EIS)

**Figure 8:**
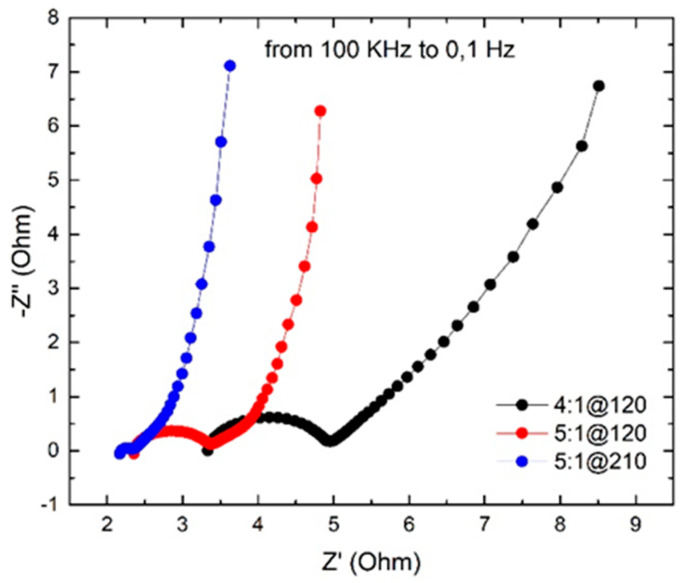
Electrochemical impedance spectroscopy (EIS) results of 4:1@120 (black), 5:1@120 (red), and 5:1@210 (blue) samples with a potential amplitude of 0.005 VRMS.

Based on the illustrative methodology described in Ref. [47], we now present the results of electrochemical impedance spectroscopy (EIS) measurements. Indeed, EIS data can effectively be visualized using Nyquist plots (Figure S6), which help assess the impedance features of electrochemical systems like batteries and supercapacitors. Our analysis of the Nyquist diagrams in Figure 8, has enabled the detailed quantification of several resistive components within our devices. This includes the electrode resistance (R_A_), identified at the high-frequency intercept on the real axis, which affects overall conductivity. The interface resistance between electrodes and electrolyte (R_BA_ = R_B_ − R_A_) reflects the charge transfer efficiency, while the electrolyte diffusion resistance within the electrode bulk (R_CB_ = R_C_ − R_B_) provides insight into the ion transport dynamics. Collectively, these resistances contribute to the equivalent series resistance (ESR) of the device, a crucial factor in determining its performance. The EIS measurements for this study were carried out with a potential amplitude of 0.005 VRMS to ensure accurate impedance characterization without perturbing the system significantly. The resistance values obtained are reported in Table S2.

The Nyquist plot and impedance values demonstrate the influence of activation conditions on the electrochemical performance of our most promising electrodes. As the activation becomes more intense (higher KOH ratio and longer duration), the electrical resistance (R_A_) of the electrodes decreases, indicating improved conductivity that is consistent with the enhanced crystallinity observed in Raman spectra (Figure 3 and Figure 4). The charge transfer resistance at the electrode/electrolyte interface (R_BA_) also decreases, suggesting better chemical affinity and wettability at more advanced activation states. The Warburg impedance (R_CB_), indicative of electrolyte ion diffusion into the electrode pores, remains similar between samples activated under more rigorous conditions (5:1@120 and 5:1@210), pointing to a stable ion diffusion environment despite variations in activation. This stability in ion diffusion is critical for supercapacitor performance and is not significantly affected by the mesopore volume changes between these samples. The ESR, a comprehensive indicator of total resistive losses, is favorably reduced in samples with higher activation, affirming the general efficiency of the supercapacitors. These observations collectively validate the superior capacitive performance of our electrodes under the specified conditions of preparation and operation.

To add further context to our study, we performed extra galvanostatic charge-discharge experiments at various current densities, as depicted in Figure S7. Notably, under high current stress (3 A/g and 5 A/g), the 4:1@120 sample was unable to sustain capacitive behavior, swiftly reaching the set voltage limit, thus preventing further cycling. This suggests a limited electrochemical rate capability, likely due to suboptimal porosity or insufficient electronic conductivity. In contrast, the 5:1@120 and 5:1@210 samples exhibited linear potential-time relationships during charge-discharge cycles, despite a marked voltage drop as the current density increased. This voltage drop, more pronounced at the highest current tested, is indicative of the ohmic drop within the supercapacitors, reflecting the internal resistance and validating their enhanced rate capabilities. Such performance is critical for applications requiring rapid charge and discharge cycles, and our results highlight the importance of optimizing activation parameters to achieve superior rate performance in supercapacitor electrodes.

## 4. A Comparison with Previous Literature

To further emphasize our findings, Table 4 presents a comparison of AC derived from different precursors, highlighting variations in SSA and specific capacitance. It is important to point out that specific capacitance is influenced by the electrolyte type and the design of the experimental cell. In a three-electrode setup, the potential is applied across a single working electrode against a stable reference electrode, providing a broader potential window for the measurement of capacitance than in a two-electrode symmetrical cell, where the potential is divided between two working electrodes [48]. Consequently, specific capacitance measurements in a three-electrode system can appear significantly higher due to the extended potential range and increased accessible surface area, often leading to reported values that can quadruple those measured in a two-electrode system under similar conditions. As an example, Ranaweera et al. [49] reported a high charge storage capacitance of 407 F/g using a three-electrode setup for orange-peel-derived electrodes.

In the literature, on the other hand, there are works where the specific capacitance obtained from a two-electrode system, as determined from CCGCD analysis, is normalized to reflect the potential outcomes in a three-electrode system. This normalization often involves adjusting for electrode mass—either by doubling the denominator to represent a single electrode mass [50] or by quadrupling the specific capacitance value to account for the larger potential window and accessible surface area in a three-electrode setup [51]. However, in our study, we have held back from such normalization. Despite its theoretical justification, we recognize that it remains an approximation and have chosen to report our findings based on direct measurements to ensure clarity and precision in our reported data.

**Table 4:** A comparison with previous works.

Precursor	SSA [m^2^/g]	Electrolyte	Cell Type	$C_{s}$ [F/g]	Ref.
Waste compact disc	1390	1 M EMIMBF_4_	Two electrodes	51	[52]
Rice Husk	770	1 M Et_4_NBF_4_	Two electrodes	19	[53]
Cotton stalk	1481	1 M Et_4_NBF_4_	Two electrodes	28.5 (114 normalized to 4 factor)	[50]
Palm oil	1704	1 M H_2_S0_4_	Two electrodes	37.25 (149 normalized to 4 factor)	[54]
Pinecone	1515	1 M Na_2_SO_4_	Two electrodes	34.25 (137 normalized to 4 factor)	[55]
Cellulose	1198	1 M Na_2_SO_4_	Two electrodes	26 (96 normalized to 4 factor)	[56]
Poultry litter	3035	1 M Na_2_SO_4_	Two electrodes	41 (164 normalized to 4 factor)	[51]
Orange Juice	2203	1 M Na_2_SO_4_	Two electrodes	56	This work

From Table 4, one can see that AC derived from Italian orange juice exhibits an SSA of 2203 m^2^/g and a specific capacitance of 56 F/g, outperforming a range of biomass-derived carbons as reported in the literature. These results, obtained without the normalization adjustments commonly applied to account for three-electrode systems, suggest that the material’s high SSA is effectively leveraged for energy storage. When compared to other precursors such as waste compact discs, rice husk, and palm oil, which required normalization factors to enhance their reported specific capacitance, our as-made AC demonstrates superior capacitance, directly measured in a two-electrode system. This indicates a significant advancement in the use of sustainably sourced materials for high-performance supercapacitors, emphasizing the effectiveness of the synthesis and activation process utilized for the Italian orange juice-derived AC.

As the last remarkable point, we would like to comment that in AC derived from Italian orange juice, we found that specific capacitance is influenced by more than just surface area. While samples with similar surface areas showed varying capacitance, this discrepancy highlights the importance of balancing pore structure and electrode wettability. High surface areas, often due to nanopores, can impede ion diffusion, crucial for diffusion-controlled capacitance. On the other hand, mesopores and macropores facilitate ion diffusion but offer limited surface area, impacting surface-controlled capacitance. Our research demonstrates that by adjusting activation parameters, we can effectively manipulate pore morphology and electrode wettability, optimizing the balance between diffusion and surface control in capacitance.

## 5. Conclusions

In summary, we have explored the potential of porous carbon materials synthesized from orange juice through a carbonization-activation process, specifically for their use in symmetric supercapacitors. Utilizing a suite of characterization techniques, including SEM, TEM, Raman spectroscopy, and XPS, alongside comprehensive electrochemical testing through cyclic voltammetry and galvanostatic charge/discharge cycles, we have validated the purely capacitive nature of these novel materials.

In particular, the carbon material activated with a KOH to precursor ratio of 5:1 for 120 min (5:1@120) displayed particularly outstanding electrochemical properties. It not only exhibited high specific capacitance but also sustained this performance over numerous cycles, indicating remarkable stability. This high capacitance was achieved without the need for the upward normalization commonly seen in the literature, underscoring the intrinsic quality of the material.

Furthermore, BET and morphological examination revealed that the 5:1@120 sample possessed an optimal meso- to microporous structure, which was likely instrumental in its superior electrochemical behavior. The high SSA identified in these samples is directly associated with efficient ion transport and charge storage capabilities, essential characteristics for effective supercapacitors. Indeed, the production method for these porous carbons, utilizing a readily available citrus byproduct, represents an eco-friendly and scalable approach to supercapacitor development. This aligns with a broader shift towards sustainable energy technologies. Hence, the important electrochemical performance coupled with a simple and green synthesis route makes the 5:1@120 activated carbon a strong contender for next-generation supercapacitor materials, potentially revolutionizing the use of bio-waste derivatives in the field of energy storage.

Finally, while the specific capacitance of approximately 56 F/g is on the lower side, our current research catalyzes future studies that could propose enhancements to boost electrochemical performance. Potential avenues for improvement include: (i) optimizing pore size and distribution by weak base-assisted hydrolysis [57], (ii) refining electrolyte selection, (iii) integrating conductive additives like carbon black or graphene [58], (iv) doping with heteroatoms such as nitrogen, boron, or sulfur [59], (v) meticulously adjusting the activation process parameters like temperature, duration, or chemical concentrations, and (vi) enhancing the packing density of the activated carbon within the electrode to potentially elevate the volumetric capacitance and the development of more complex systems such as gel polymer electrolytes (GPEs) with high ionic conductivity [60].

## Figures and Tables

**Table 1:** Specific surface areas (m^2^/g) using Brunauer–Emmett–Teller (BET) analysis for different samples under study.

	Activation Time (min)		
Ratio (w%)	30	120	210
3:1	1271.58 ± 14.22	1590.11 ± 7.75	1779.68 ± 8.91
4:1	1865.69 ± 12.95	2195.85 ± 7.66	1573.91 ± 6.26
5:1	1309.97 ± 12.64	2202.81 ± 7.43	1926.69 ± 7.42

**Table 2:** Intensity ratio of D/G bands and 2D/G bands for different samples under analysis.

Sample	I_D_/I_G_	I_2D_/I_G_
4:1@120	0.97	---
5:1@120	0.85	0.44
5:1@210	0.68	0.60

**Table 3:** Summary of XPS bands of 5:1@120.

	**C 1s**		
C=C	284.10	46%	R^2^ = 0.999
C-OH	285.52	34%	
C=O	287.10	13%	
O-C=O	289.00	7%	
	**O 1s**		
O-C=O	531.19	15%	R^2^ = 0.999
C=O	532.82	30%	
C-O	534.55	55%	

## Data Availability

Data are contained within the article.

## Supplementary Materials

The following supporting information can be downloaded at: https://www.mdpi.com/article/10.3390/nano14010071/s1. Figure S1. (a) Nitrogen sorption isotherms of all active carbons, showing some hysteresis. (b) Pore size distributions of all active carbons obtained by the NLDFT method. Figure S2. SEM image of pyrolyzed sample. Figure S3. Microsphere Size Distribution after pyrolysis. Average diameter 6.6 μm with a standard deviation of 1.5 μm. Figure S4. XRD pattern of a biochar sample after carbonization. Figure S5. (a) SEM and (b) TEM morphology of the AC sample (5:1@120) after long-term cycling (5000 cycles). Figure S6. Illustration of the electrochemical impedance spectroscopy (EIS) Nyquist plot. Figure S7. Galvanostatic cycling for different current densities: (a) 0.5 A/g, (b) 1.0 A/g, (c) 3.0 A/g, and (d) 5 A/g. Table S1. Position of Raman peaks for different samples under analysis. Table S2. Resistance values obtained from electrochemical impedance spectroscopy (EIS) measurements.
