# Supplementary material for: Stable Supercapacitors Based on Activated Carbon Prepared from Italian Orange Juice
Source: Nanomaterials (Basel). 2023 Dec 26;14(1):71. doi: 10.3390/nano14010071 (PMC10780622; doi:10.3390/nano14010071)
Supplement: Supplementary file 1 [file nanomaterials-14-00071-s001.zip › nanomaterials-2759617-SI.pdf]

nanomaterials

Article

Stable supercapacitors based on activated carbon prepared from Italian orange juice

Andrea Scarcello[1,2], Francesca Alessandro[3], Yolenny Cruz Salazar[1,2], Melvin Arias Polanco[2,4], Cristian Vacacela Gomez[2,5], Talia Tene[2,6], Marco Guevara[7], Stefano Bellucci[5], Salvatore Straface[8] and Lorenzo S. Caputi[1,2]

[1]Surface Nanoscience Group, Department of Physics, University of Calabria, 87036 Rende, Cosenza, Italy
[2]UNICARIBE Research Center, University of Calabria, Rende, I-87036, Italy
[3]Institute on Membrane Technology, National Research Council of Italy (CNR-ITM), Via P.Bucci 17/C, 87036 Rende, Italy
[4]Laboratorio de Nanotecnología, Area de Ciencias Básicas y Ambientales, Instituto Tecnológico de Santo Domingo, Av. Los Próceres, Santo Domingo, 10602, Dominican Republic
[5]INFN-Laboratori Nazionali di Frascati, I-00044 Frascati, RM, Italy
[6]Department of Chemistry, Universidad Tecnica Particular de Loja, Loja, 110160, Ecuador
[7]Faculty of Mechanical Engineering, Escuela Superior Politécnica de Chimborazo (ESPOCH), Riobamba, 060155, Ecuador
[8]Department of Environmental Engineering (DIAm), University of Calabria, Via P. Bucci, Cubo 42B, Rende, I-87036, Italy

* Correspondence: A.S, Email: andrea.scarcello@unical.it; L.S.C, Email: lorenzo.caputi@fis.unical.it

Supplementary Figures

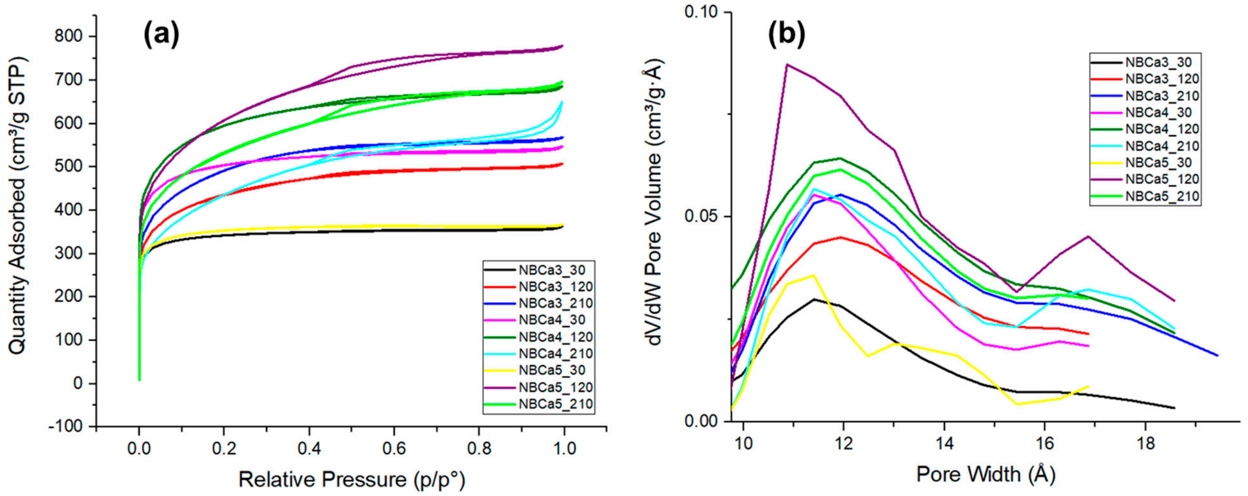

Figure S1. (a) Nitrogen sorption isotherms of all active carbons, showing some hysteresis. (b) Pore size distributions of all active carbons obtained by the NLDFT method.

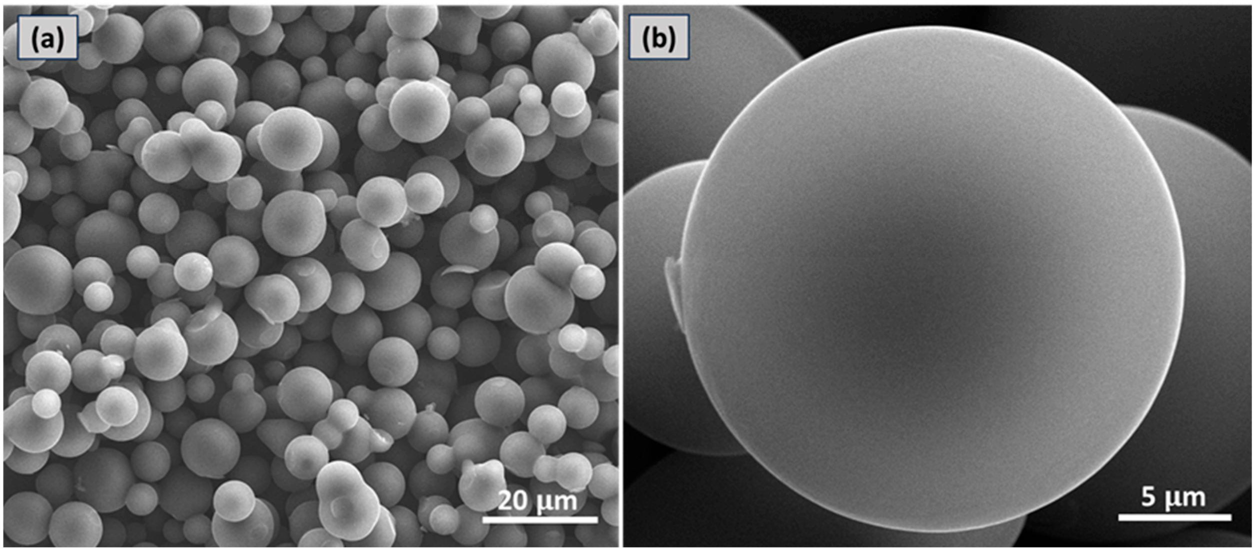

Figure S2. SEM image of pyrolyzed sample.

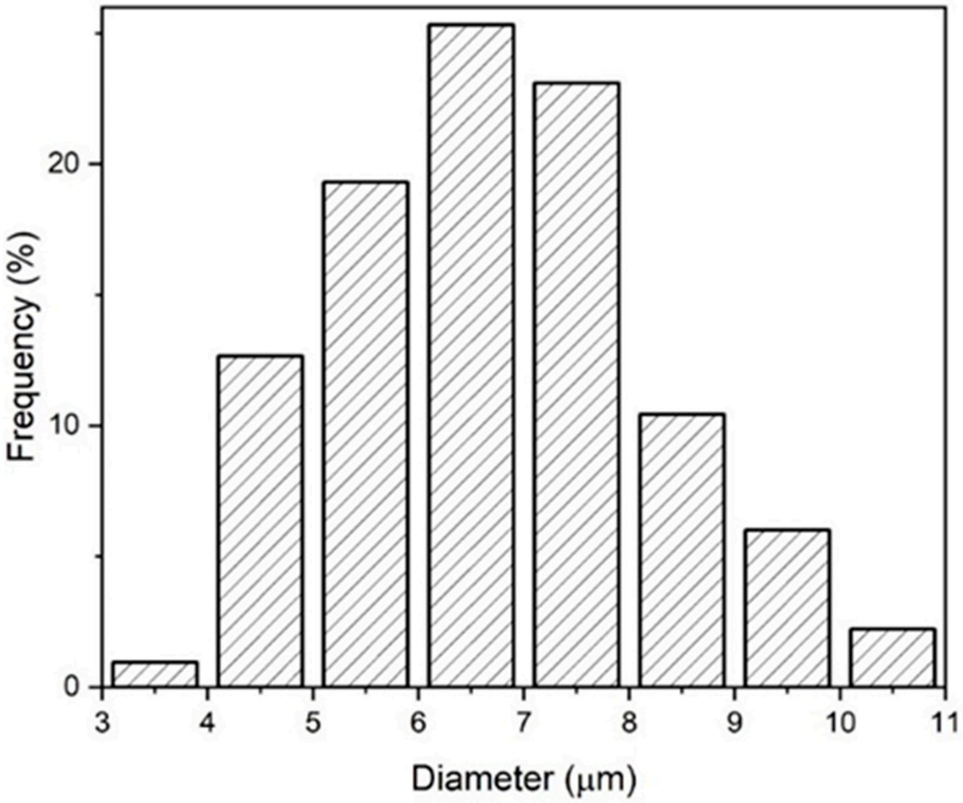

Figure S3. Microsphere Size Distribution after pyrolysis. Average diameter 6.6 μm with a standard deviation of 1.5 μm.

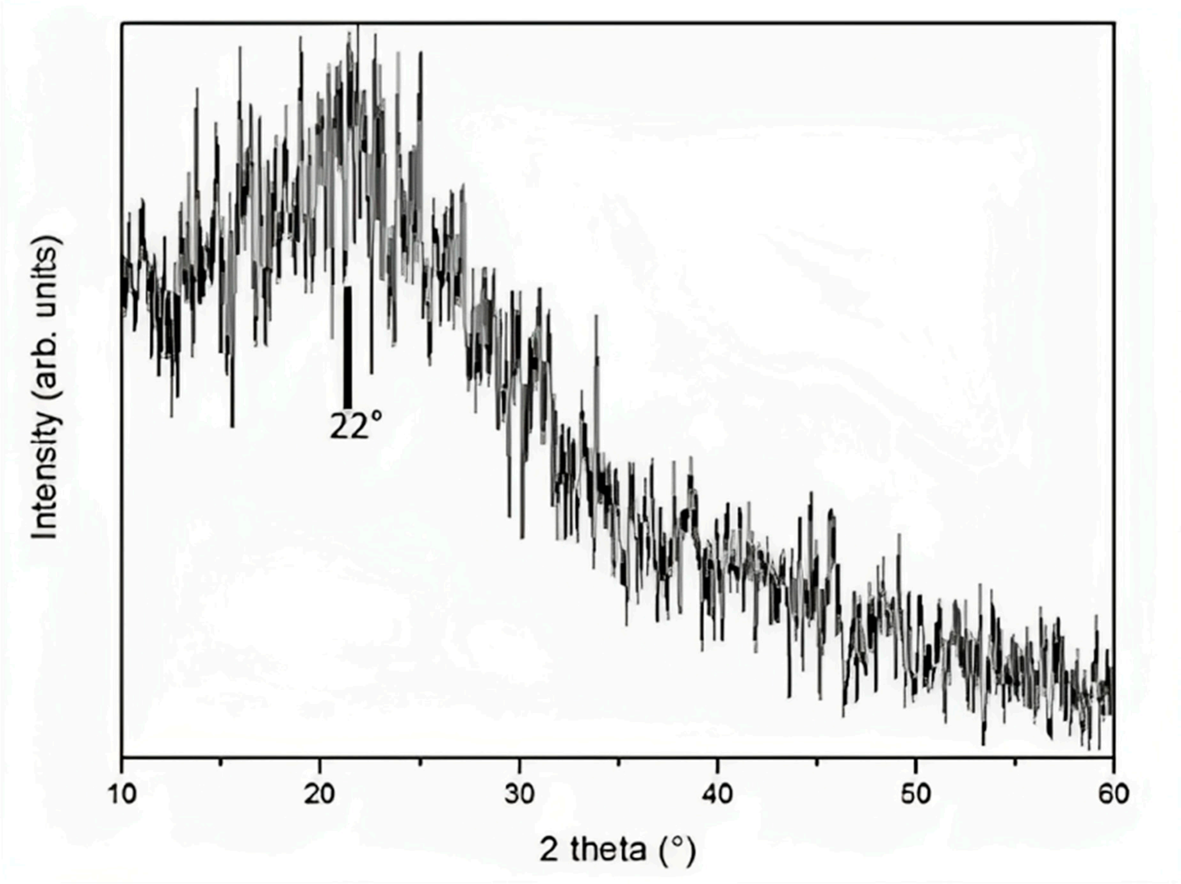

Figure S4. XRD pattern of a biochar sample after carbonization.

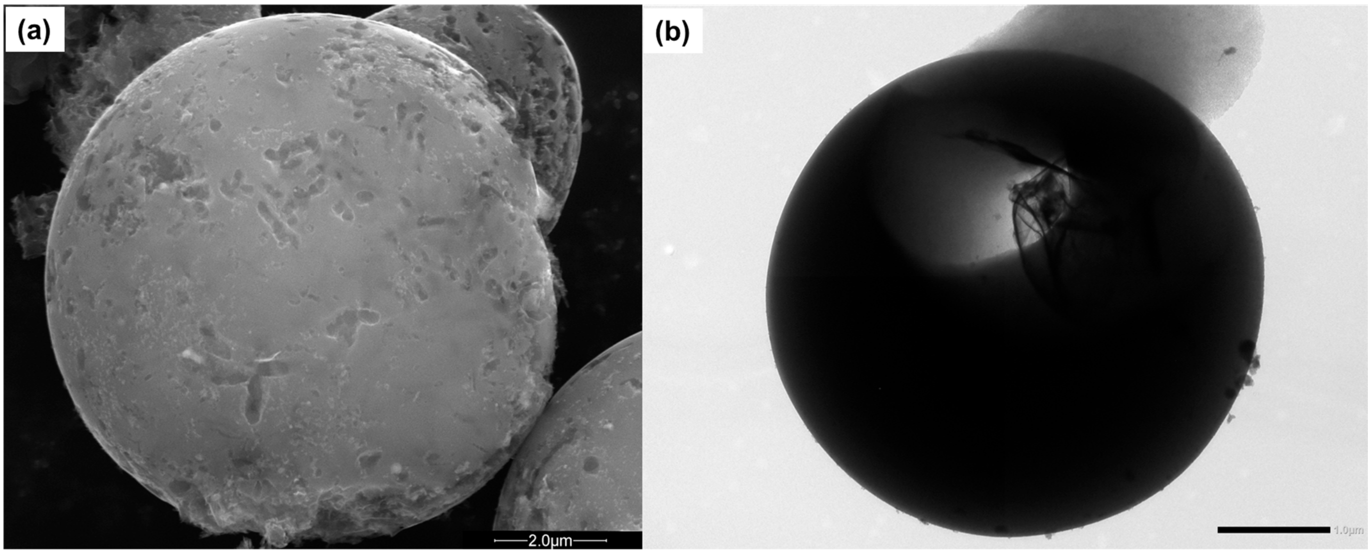

Figure S5. (a) SEM and (b) TEM morphology of the AC sample (5:1@120) after long-term cycling (5000 cycles).

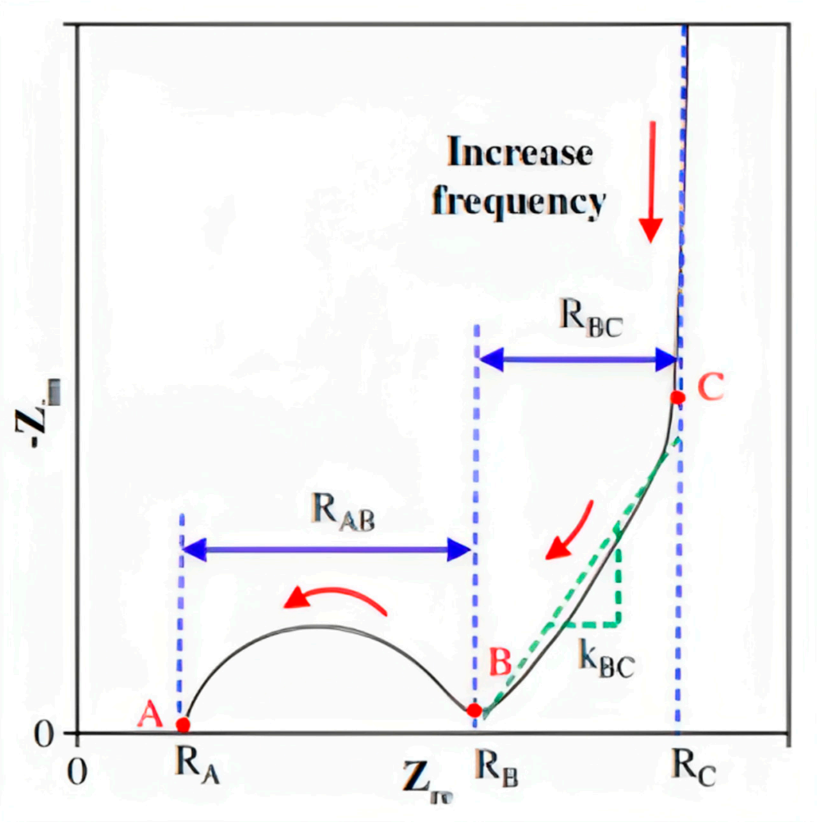

Figure S6. Illustration of the electrochemical impedance spectroscopy (EIS) Nyquist plot.

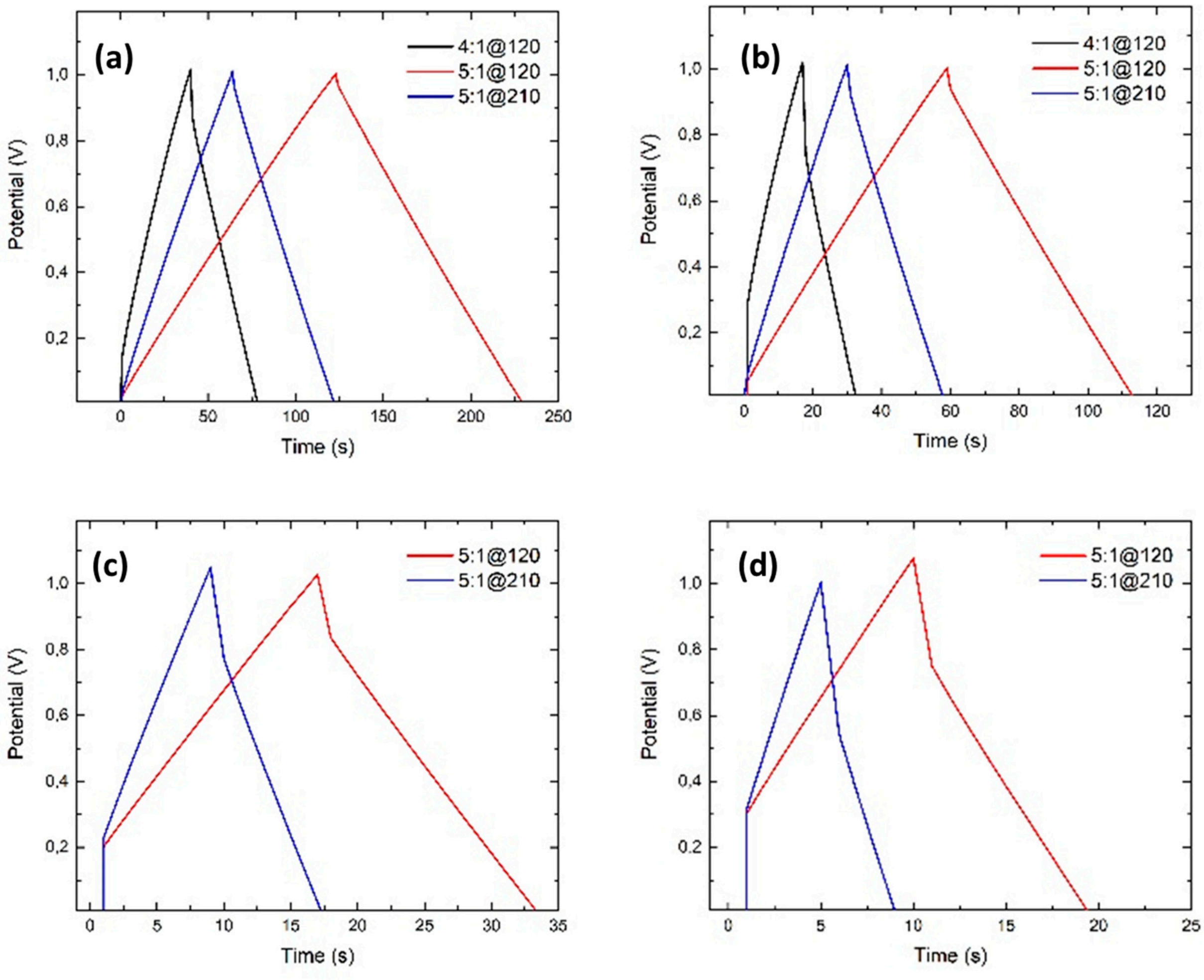

Figure S7. Galvanostatic cycling for different current densities: (a) 0.5 A/g, (b) 1.0 A/g, (c) 3.0 A/g, and (d) 5 A/g.

Supplementary Tables

Table S1. Position of Raman peaks for different samples under analysis.

Sample	D* (cm^{-1})	D (cm^{-1})	D** (cm^{-1})	G (cm^{-1})	2D (cm^{-1})	R^2
4:1@120	1198.91	1333.47	1439.41	1579.53	---	0.971
5:1@120	1221.78	1336.89	1431.97	1582.21	2678.37	0.997
5:1@210	1227.72	1335.71	1418.60	1574.74	2674.45	0.978

Table S2. Resistance values obtained from electrochemical impedance spectroscopy (EIS) measurements.

Devices	RA [Ω]	RB [Ω]	RC [Ω]	RBA [Ω]	RCB [Ω]	ESR [Ω]
4:1@120	3.33	4.96	6.85	1.63	1.89	7.83
51@120	2.36	3.38	3.83	1.02	0.45	4.41
5:1@210	2.17	2.33	2.79	0.16	0.46	2.98
